# Endoscopic submucosal dissection for superficial esophageal cancer with ulcer scarring using a combination of pocket creation, gel immersion, and red dichromatic imaging

**DOI:** 10.1055/a-2234-8435

**Published:** 2024-01-30

**Authors:** Tsubasa Ishikawa, Tomoaki Tashima, Takahiro Muramatsu, Yumi Mashimo, Shomei Ryozawa

**Affiliations:** 1 183786 Department of Gastroenterology, Saitama Medical University International Medical Center, Hidaka, Japan

In recent years, the usefulness of endoscopic resection (ER) methods, such as underwater endoscopic submucosal dissection, has been widely reported 1 . Moreover, recently, from the viewpoint of securing the visual field during endoscopy, gel immersion endoscopy has been reported to be useful 2 3 . Gel immersion endoscopy and underwater ER are used in challenging endoscopic procedures owing to their buoyancy effects. The pocket creation method is widely used to overcome endoscopic submucosal dissection (ESD) difficulties 4 . Red dichromatic imaging, which improves the visibility of deep blood vessels and bleeding points using longer wavelengths of light, is recently being extensively used, and its effectiveness has been reported 5 . We used red dichromatic imaging to improve visibility of the submucosal and muscular layers during ESD.

Here, we describe a case of successful ESD of a superficial esophageal carcinoma on a scar after ER using the pocket creation method, underwater conditions, gel immersion endoscopy, and other techniques.

A 68-year-old man, who had previously undergone curative resection of esophageal cancer in the middle thoracic region using ESD 30 years prior, was referred for follow-up. An upper gastrointestinal endoscopy revealed a new superficial carcinoma of the esophagus (30 mm in size) located on the post-treatment scar ( Fig. 1 ). ESD was planned. During this treatment, in addition to the pocket creation method to break through the fibrosis, underwater endoscopic submucosal dissection and gel immersion endoscopy devices were used inside the pocket to add to the buoyancy effect ( Fig. 2 , Fig. 3 ). Red dichromatic imaging was also used to visualize the fibrosed area and to ensure a good visual field ( Fig. 4 ). These measures made it possible to complete submucosal dissection with clear visibility of the fibrosed area ( Video 1 ).

**Fig. 1:**
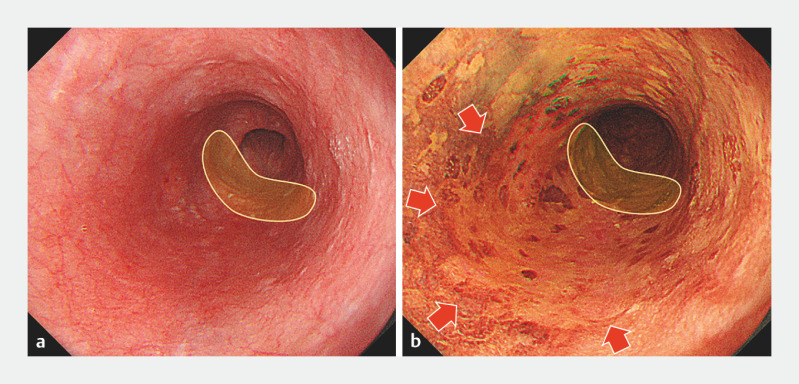
The target lesion. The lesion was recognized as a brownish area, 30 mm in size, on the posterior wall, 35 cm from the incisor row. The area covered by the yellow enclosure indicates the scar from previous treatment, and the red arrow indicates the target lesion. **a** White light imaging. **b** After spraying with Lugolʼs solution, the lesion was observable in the Lugolʼs voiding area.

**Fig. 2:**
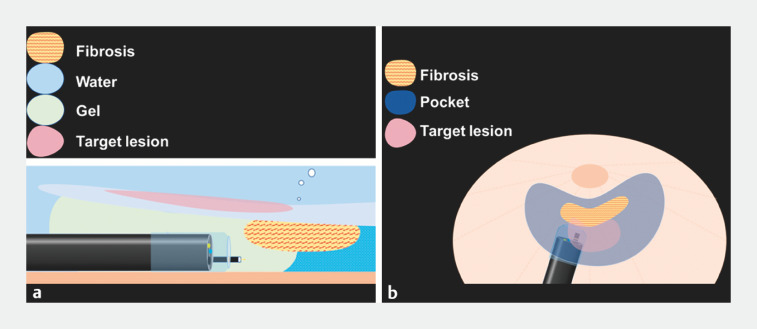
Schema image of the treatment process. **a** Fibrosis due to previous treatment was considered to have formed in the shallow submucosal layer, and the visibility of the fibrosis was ensured through filling the pocket with gel. **b** Using the pocket creation method, submucosal dissection was performed to a greater extent beyond the lesion border to break through the fibrosis.

**Fig. 3:**
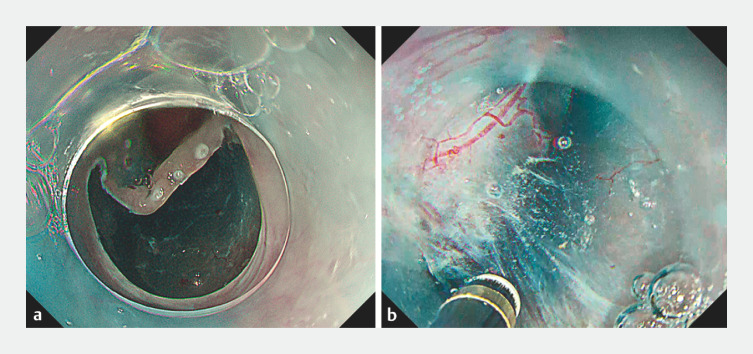
Pocket creation method. **a** Mucosal incision was initiated from the oral side of the lesion, and a pocket was created. **b** Inside the pocket, the gel provided a good visual field.

**Fig. 4:**
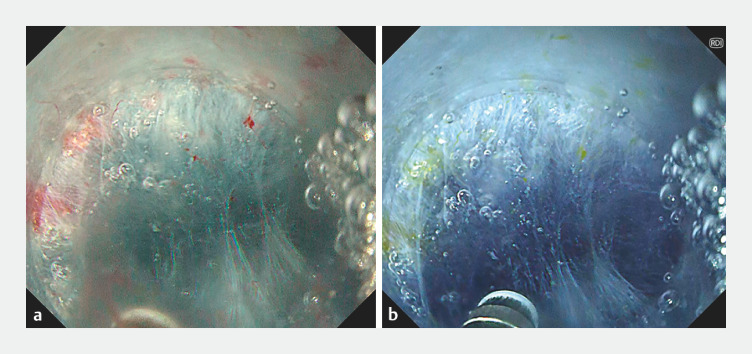
Red dichromatic imaging. **a** White light observation may cause gels and water to become cloudy. **b** Red dichromatic imaging, using a long wavelength band that is not easily scattered or absorbed proximally, improves target visibility.

Successful pharyngeal endoscopic submucosal dissection using a novel clip-traction band device.Video 1

Endoscopy_UCTN_Code_TTT_1AO_2AC

## Notes

Endoscopy E-Videos https://eref.thieme.de/e-videos
*E-Videos* is an open access online section of the journal *Endoscopy* , reporting on interesting cases and new techniques in gastroenterological endoscopy. All papers include a high-quality video and are published with a Creative Commons CC-BY license. Endoscopy E-Videos qualify for HINARI discounts and waivers and eligibility is automatically checked during the submission process. We grant 100% waivers to articles whose corresponding authors are based in Group A countries and 50% waivers to those who are based in Group B countries as classified by Research4Life (see: https://www.research4life.org/access/eligibility/ ). This section has its own submission website at https://mc.manuscriptcentral.com/e-videos .
